# Interaction Mechanisms of Cavitation Bubbles Induced by Spatially and Temporally Separated fs-Laser Pulses

**DOI:** 10.1371/journal.pone.0114437

**Published:** 2014-12-11

**Authors:** Nadine Tinne, Brigitte Kaune, Alexander Krüger, Tammo Ripken

**Affiliations:** Laser Zentrum Hannover e.V., Biomedical Optics Department, Hannover, Germany; UC San Diego, United States of America

## Abstract

The emerging use of femtosecond lasers with high repetition rates in the MHz regime together with limited scan speed implies possible mutual optical and dynamical interaction effects of the individual cutting spots. In order to get more insight into the dynamics a time-resolved photographic analysis of the interaction of cavitation bubbles is presented. Particularly, we investigated the influence of fs-laser pulses and their resulting bubble dynamics with various spatial as well as temporal separations. Different time courses of characteristic interaction effects between the cavitation bubbles were observed depending on pulse energy and spatio-temporal pulse separation. These ranged from merely no interaction to the phenomena of strong water jet formation. Afterwards, the mechanisms are discussed regarding their impact on the medical application of effective tissue cutting lateral to the laser beam direction with best possible axial precision: the mechanical forces of photodisruption as well as the occurring water jet should have low axial extend and a preferably lateral priority. Furthermore, the overall efficiency of energy conversion into controlled mechanical impact should be maximized compared to the transmitted pulse energy and unwanted long range mechanical side effects, e.g. shock waves, axial jet components. In conclusion, these experimental results are of great importance for the prospective optimization of the ophthalmic surgical process with high-repetition rate fs-lasers.

## Introduction

Nowadays, in numerous therapeutic applications of ophthalmic laser surgery the fundamental physical effect of photodisruption is used for tissue dissection. Tight focusing of an ultra-short laser pulse leads to nonlinear absorption processes at the focal volume due to the high intensities [1]–[3]. A dense free electron plasma is generated which results in a laser-induced optical breakdown (LIOB) for exceeding the critical electron density in the order of ρ_cr_ = 10^21^ cm^−3^ [3]. The energy is transferred to the atomic system via recombination and collision effects which lead to a fast increase of temperature as well as pressure. As a consequence of this pressure rise a shock wave propagates into the surrounding medium; its tensile stress component results in cavitation bubble formation by exceeding the spinodal limit far below the critical point of water [3]–[4]. The oscillation of the cavity mechanically ruptures the tissue surrounding the focal spot. By scanning the laser focus beyond the surface and stringing together the subsequent single laser foci the medium can be manipulated in any three-dimensional pattern. The fundamental interaction effect after focusing a single ultra-short laser pulse into an aqueous medium like transparent biological tissue has already been studied extensively; various former publications deal with its explicit description (see for example [1], [3], [5]–[11]).

Only for the regime of low-repetition rate laser systems the description of the photodisruptive cutting process by an isolated single pulse event might remain true. At the onset of refractive surgery, the first clinical fs-laser systems disposed a relatively high pulse energy (>1 µJ) at a comparably low repetition rate (kHz regime) [12]. However, a steady decrease in the applied laser pulse energy can be denoted in the course of time [12]–[13]. This evolution has led to a significant enhancement in treatment accuracy on the one hand. The reason is that the resulting maximum cavitation bubble radius and hence the damaged volume scale with the applied pulse energy [2]–[3]. On the other hand, for retaining the duration of treatment the repetition rates of the clinical systems have increased simultaneously [12]–[13].

At pulse energies close to the LIOB threshold and repetition rates in the range of some 10 kHz subsequent laser pulses and the previously induced cavitation bubbles can hardly interact with each other. The lifetime of a cavitation bubble T_c_ as well as its maximum bubble radius R_max_ depend on the applied laser pulse energy E_pulse_ [3]. Close to the breakdown threshold the lifetime reaches only a value in the lower microsecond regime [14]. Unfortunately, the previous pulse's cavitation bubble may still oscillate while focusing a next pulse in its vicinity with increasing repetition rate. Assuming a bubble lifetime of about 3 µs, even repetition rates of 300 kHz and higher would imply a temporal overlap of the two cavitation bubbles' oscillations. Therefore, the interaction between oscillating cavitation bubbles and subsequent temporally as well as spatially separated laser pulses or the appropriate cavities becomes relevant for high-repetition rate fs-laser systems. The well-known single bubble cutting process described above may be modified due to an affection of the subsequent laser pulse's LIOB effect. The potential mechanisms of this pulse-to-pulse interaction are of tissue-optical and fluid-mechanical nature: a change of optical nonlinear absorption and conversion efficiency as well as of the amount of energy transmission, refraction and defocusing of the laser beam at the first bubble's surface are the optical interaction effects; jet formation and penetration of surrounding tissue the mechanical ones. Before a decision can be made which of them are disadvantageous or which are even desirable and utilizable for the cutting process a profound analysis has to be made. This should be performed regarding the parameters leading to the different phenomena, their scalability and reproducibility in water and the transfer to real organic tissue.

Up to now, different constitutional time-resolved studies of subsequently focused laser pulses and cavitation bubbles have been published: On the one hand the interaction effects of only spatially separated cavitation bubbles were analyzed [15]–[25]. It is associated with an asymmetric oscillation behavior as well as a formation of a strong jet perpendicular to the optical axis of the laser [15]. On the other hand an only temporal separation of laser pulses was investigated which leads to a scenario of focusing a subsequent laser pulse into an existing cavitation bubble [26]–[27]. In this case, a further LIOB would be suppressed due to the increased breakdown threshold for water vapor inside the cavity. Hence, there is an increase of laser transmission behind the focal volume [27]. Furthermore, certain US patents deal with two simultaneously generated cavitation bubbles, which are utilized for surface processing (for example [28]).

The emphasis of the experiments presented here is the characterization of the fundamental interaction effects of two or more cavitation bubbles or fs-laser pulses, respectively, which have a various temporal as well as spatial separation. It is of great interest for estimating of collateral damage during medical treatment. Furthermore, there is a possibility of a prospective optimization of the surgical process with high-repetition rate fs-laser pulses. The investigation of the resulting cavitation bubble dynamics and the interaction mechanisms was realized by time-resolved photography. It is a well-established method of analyzing this effect of disruptive laser-tissue interaction [2]–[3], [6], [29]–[30].

As we will show in the following sections, a customized laboratory fs-laser setup allowed us to vary the overlap between pulses and bubbles over a wide range. As a result we will present 11 different interaction mechanism within this parameter range. These interaction scenarios will be discussed regarding their possible merit and usability in ophthalmic laser surgical systems.

## Materials and Methods

**Figure 1:**
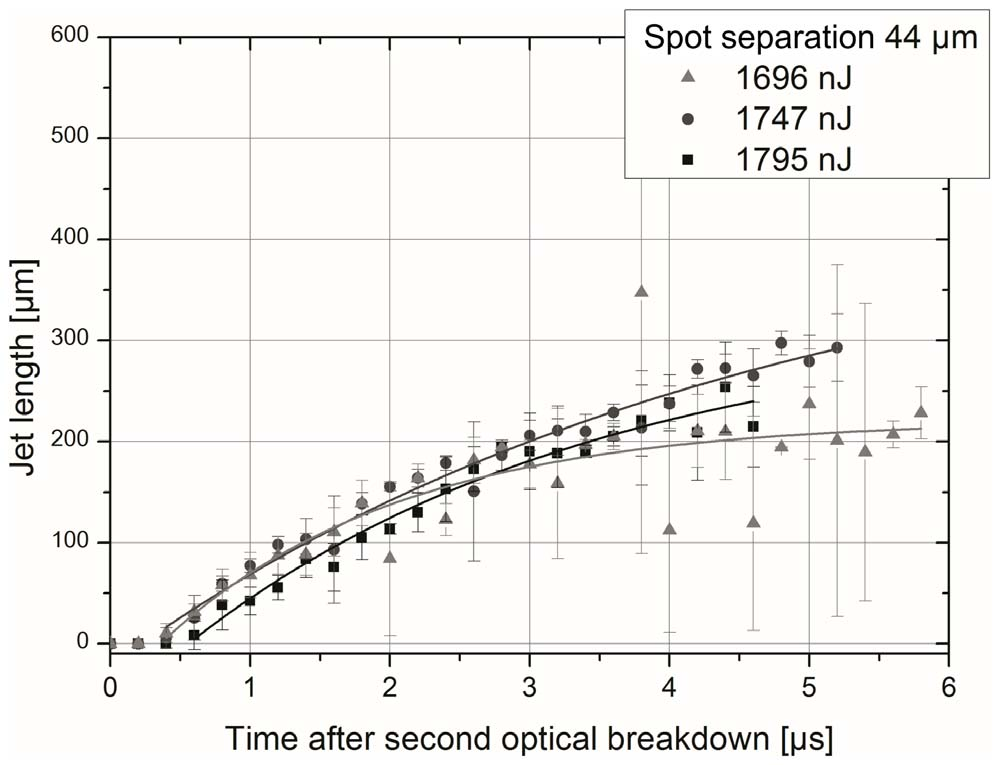
Schematic depiction of the experimental setup of the laser path (red) and the illumination path (orange). Single pulses of the fs-laser are selected by an acousto-optic modulator (AOM), half-wave plate and polarizing beam-splitter cube allow for laser power adjustment. Subsequent laser pulses are spatially separated via polygon scanner and a Keplerian telescope imaging (see also magnified image detail). The focal region inside the sample medium-filled cuvette is illuminated homogeneously by Koehler illumination and a magnified image of the cavitation bubble is reproduced on the chip of the CCD camera.

The experimental setup which was an installation for time-resolved photography of the cavitation bubble dynamics can be divided in two light paths; it is shown schematically in Fig. 1. The red one is the path of the fs-laser beam with finally focusing it into a cuvette in order to create the LIOB. The orange beam is used for illumination as well as imaging of the cavitation bubbles.

### Focusing of separated fs-laser pulses and cavitation bubble generation

The light path for the laser beam is shown in Fig. 1. It was used for laser power adjustment and expansion if beam diameter between the laser system and the spot of LIOB. Additionally, the setup was especially designed for realizing various scenarios of temporal and spatial pulse overlap. The experiments for analyzing the bubble-bubble interaction were performed with the fs-laser system “µJewel” by *IMRA America Inc.* (Ann Abor, USA) with central wavelength λ = 1040 nm, pulse width τ = 389 fs, and repetition rate f_rep_ = 100 kHz.

For an external triggered pulse picking of a defined number of laser pulses, the laser beam was coupled into an acousto-optic modulator (AOM) first. The following optical elements were a half-wave plate and a polarizing beam splitter cube for energy attenuation as well as a variable beam expander. Following this, the laser beam passed through the line scanner setup. The angular spacing for subsequent laser pulses was generated by a polygon scanner with 24 mirror facets (*Kugler GmbH*, Salem, Germany). A Keplerian telescope (see lenses L1 and L2 in Fig. 1) in combination with a periscope type mirror pair (M3 and M4) projected a vertical orientated optical image of the scanner mirror surface on the back focal aperture of the laser-focusing microscope objective. Hence, it resulted in a translation of the angular beam deflection into a lateral focus separation which is located within the Koehler-illuminated plane.

The laser beam was focused into a cuvette by the immersion-free microscope objective (NA = 0.65, *Olympus GmbH*, Hamburg, Germany) with a maximum cover slip correction of 1.2 mm. The entrance aperture of the focusing objective was illuminated and filled with the 1/e^2^-beam diameter of the Gaussian laser beam profile. The maximum length of the scanning line within the cuvette amounted to about 1380 µm; for a typical scanning width of up to 150 µm the energy loss due to vignetting was less than 0.35%. The 10×10 mm^2^ standard fluorescence cuvette (wall thickness of 1.25 mm; *Hellma GmbH & Co. KG*, Müllheim, Germany) filled with the sample medium was mounted on a motorized micrometer 3D-translation stage (*Physik Instrumente GmbH & Co. KG*, Karlsruhe, Germany) which could be moved relatively to the focus after every laser pulse application and acquisition of a short time photograph. Thus, in more solid but still aqueous sample media a potential influence of a previous pulse's persistent mechanical damage at the focal spot on the analyzed effects could be avoided.

#### Sample Medium

At first, de-ionized water was used as a sample medium for the transparent tissue of the crystalline lens or the cornea. Various publications have shown that the optical and thermodynamic properties of water also determine the LIOB process and cavitation bubble occurrence in highly hydrated tissues like e.g. cornea [2]–[3], [6], [29]–[30]. However, the rheological properties of tissue as an aqueous more solid medium differ strongly and had to be taken into account. For that reason, selected experiments for analyzing the resulting bubble oscillation due to two spatial as well as temporal separated fs-laser pulses were performed in different concentrations (1%, 2%, and 5%) of porcine gelatin (*Sigma Aldrich Chemie GmbH*, Taufkirchen, Germany) mimicking the viscosity of biological tissue (as before agar gel in [31]–[32] or PAA in [33]–[34]). The rheological properties of the solutions changed with higher gelatin concentration: While the 5% composition was almost an aqueous solid, the 1% gelatin-water solution was still kind of colloidal; a determination of viscosity was not possible. Furthermore, porcine vitreous body obtained from a local slaughterhouse (*Schlachthof Hannover*, Hanover, Germany) was used as another sample medium.

#### Time-resolved photography

Time-resolved photography as experimental procedure allows for analyzing very fast dynamic phenomena like oscillating cavitation bubbles with a lifetime of some microseconds; its basic principal is sectioning the process into specific events. The method of time-resolved photography as well as this second optical path of the experimental setup can be found explicitly described in former publications [15], [27]. Briefly, the light of an external triggered flash lamp (*High-Speed Photo Systeme*, Wedel, Germany) was collimated onto the cuvette (Koehler type illumination) and the shadow contrast of the cavitation bubble was imaged on a charged coupled device (CCD) chip of the camera (*Lumenera*, Ottawa, Canada) using a long working distance microscope objective (20x, NA = 0.28; *Mitutoyo*, Kawasaki, Japan). The flash lamp allowed for illumination times as short as 17.43±0.55**ns (full width at half maximum) with a jitter of ≤50**ns. The optical resolution of the imaging system was about 2.32 µm, while the experimentally determined magnification was 19.

A delay generator was utilized for realizing the controlling and timing (*Bergmann Messgeräte Entwicklung KG*, Murnau, Germany; for details see [15], [27]). Due to the implementation of the polygon scanner in the laser beam path a modification of the input signal was performed. By this means, the reproducibility of the observed effect regarding the focus position within the sample medium had to be ensured. Therefore, a logic electronic circuit was developed: By processing the laser trigger signal and the encoder signal of the scanner using an AND-gate the pulse picking and the time-resolved measurement was only performed with laser pulses which hit the scanner within a tolerance period of 2 µs after reaching its middle position. Thus, the focal position could be determined with an accuracy of Δx = 2 µs · v_scan_; the scanning velocity of the focal spot v_scan_, in turn, could be continuously chosen between 2.5 µm/µs and 16.0 µm/µs. Consequently, the bubbles always appeared inside the cuvette within the field of view of the camera. The variance of the scanning angle between two subsequently taken pictures and hence the ultimate focus position within the medium scaled with the actual scanning velocity. The characterization of this behavior showed a maximum deviation of the focal spot of 25 µm at a scanning velocity of 13 m/s, while typical velocities lied only in the range of 3–6 m/s.

The analysis of the individual images was performed by using the open source software *ImageJ*. The procedure implied an identification of the bubble contour and least ellipsoidal fitting to the contour to determine the axial as well as radial bubble diameter. Furthermore, the distance between the geometric centers of mass of two bubbles could be determined. During evaluation of experimental results the spot separation was identified by means of scanning velocity; for this purpose, the velocity was characterized previously regarding its dependency on the spot separation via image analysis. In the end, the bubble radii or the spatial separation were calculated by considering the imaging system's magnification of about 19. It was calculated from the equivalent object sampling size of 1 px = 0.34 µm as well as the CCD chip pixel width of 6.45 µm. This ratio was determined experimentally by traversing a needle within the Koehler-illuminated plane via a micrometer stage.

#### Experimental procedure

The energy threshold for the generation of a LIOB is characteristic for the experimental setup. Hence, it was important for the comparison with other publications as well as for evaluation and normalization of effects scaling with the applied laser pulse energy. Within the fs-regime, detection of a cavitation bubble (here with a spatial imaging resolution of about 2.32 µm) is the most reliable criterion to determine the breakdown threshold energy [3], [9], [35]. Here, the threshold was defined as the laser pulse energy at which almost each pulse leads to a visible cavitation bubble as a result of an optical breakdown (>90% probability).

Analyzing only two laser pulses with a defined temporal as well as spatial separation provided a simplified model of the fundamental bubble-pulse or even bubble-bubble interaction mechanisms during laser surgery. In principal, these effects depended on two parameters: (1) the temporal and (2) the spatial separation. They were varied in the study presented here. Parameter (1) represented the first bubble's oscillation phase at the point of time when a subsequent laser pulse is focused nearby. In turn, parameter (2) demonstrated the actual distance between the surface of the previous bubble and the second focal spot.

The latter one was directly operated by variation of scanning velocity during the experimental procedure. However, the different temporal pulse overlap was realized indirectly by variation of the pulse energy for the repetition rate of the “µJewel” laser system was restricted to 100 kHz. As mentioned above, the cavitation bubble lifetime T_c_ as well as its maximum radius R_max_ scale with the applied laser pulse energy: R_max_∼T_c_ and R_max_∼E_Cav_^(1/3)^ [3]; the cavitation bubble energy E_Cav_ in turn depends on the laser pulse duration and deposited energy [35]. For this reason, the applied laser pulse energy and with this the resulting bubble life time compared to the limited temporal pulse separation Δt was scaled.

The parameter space of focus separation Δr and applied pulse energy in multiple of breakdown threshold E_th_ in water was analyzed by choosing a constant scanning velocity and hence spot separation. The pulse energy was increased continuously while the occurring interaction effects were observed via the live camera picture as well documented as image series of the resulting cavitation bubble dynamics. The pulse energy belonging to a significant change in observable interaction mechanism by showing different characteristic effects was detected via energy meter; there was an averaging over 50 laser pulses. For taking image series of a cavitation bubble dynamics there was a temporal resolution (change in delay between the pictures) of 100 ns for the experimental study presented here, whereas three pictures were taken at one point in time to statistically confirm the observed results.

Here, the jet length was measured after its breaking through the opposite cavitation bubble wall. By measuring the actual jet length within every single picture of a series the variation of length in the course of time (step size 200 ns) was determined as velocity of the jet. It was averaged over two replicates per time step and fitted by an asymptotical function (see example in S1 Figure). Hence, the maximum jet velocity achieved right after jet formation could be analyzed with an accuracy of about ±30% as an estimation from above. This comparably large standard deviation results from the variation of the exact bubble collapse time. In most of the cases, the standard deviation was far smaller.

**Figure 2:**
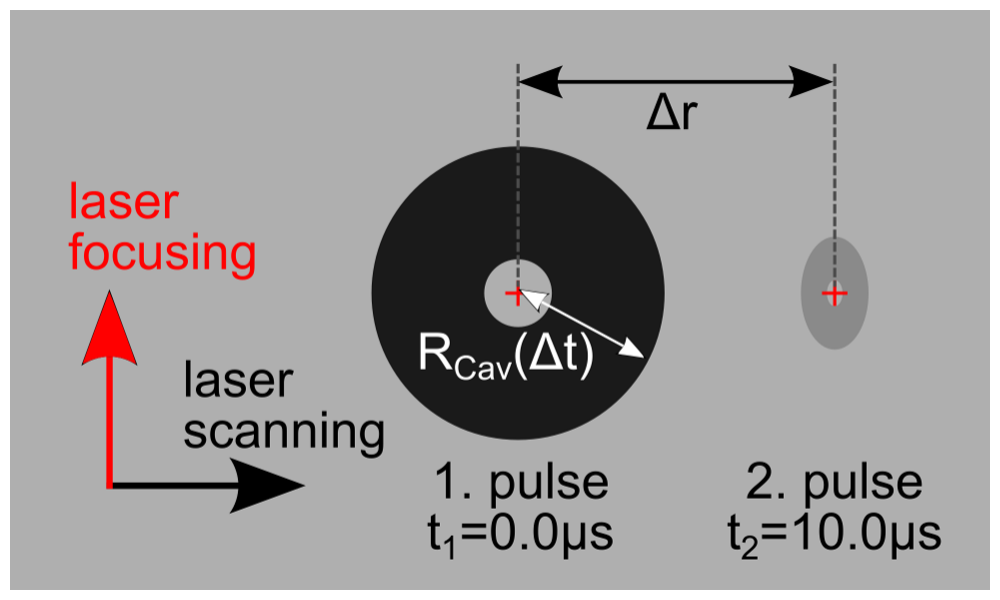
Schematic depiction of the adjustment of subsequent generated cavitation bubbles. The first bubble is induced on the left side at a time defined as t_1_ = 0.0 µs. The second cavity is generated at t_2_ after a constant delay of 10.0 µs. The bubble size is characterized as its radius R_Cav_ while the distance between the focal spots amounts to Δr.

A schematically depiction of the experimental scenario is shown in Fig. 2: The laser was scanned from left to right in each of the following pictures of the interaction mechanisms between a cavitation bubble and a subsequent laser pulse or even its cavitation bubble. Furthermore, the laser light was focused from below. The first cavitation bubble on the left side was created at a time defined as t_1_: = 0.0 µs and its radius is R_Cav_(t). At a time t_2_, which was always t_2_ = 10.0 µs in this analysis, the second pulse was focused at a certain spatial distance Δr of the first focus.

Furthermore, for approximating the medical pulse application scenario a pulse train of up to five subsequent laser pulses was applied into water in the same way for analyzing the effects of the multi-pulse or even multi-bubble interaction mechanisms, respectively. Here, the basic experimental scenario was equal to the two-bubble interaction analysis; the time delay between the other subsequent laser pulses stayed Δt = 10.0 µs.

Each series of measurement was analyzed by evaluating the bubble radius at a particular time as well as the bubble lifetime. Based on these results the actual overlap of the first pulse's cavity and the subsequent laser pulse was quantified; this procedure conduced to an optimum comparability of the following results among one another. Additionally, it allowed for discussing the results regarding pulse energies close to the LIOB threshold. Therefore, the dimensionless temporal overlap parameter η_t_ was defined as the ratio of pulse separation Δt = t_2_ - t_1_ = 1/f_rep_ to cavitation bubble lifetime T_c_:
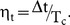
(1)


Corresponding to the value of the temporal overlap parameter the following scenarios will appear: (i) η_t_<1: The subsequent laser pulse impinges the sample medium during the first oscillation cycle of the existing cavitation bubble and (ii) η_t_>1: The second laser pulse is focused after the first collapse of the previous cavity.

The dimensionless spatial overlap parameter η_r_ was defined equivalently as the ratio of the focus separation Δr and to the actual radius of the first bubble R_Cav_(Δt):
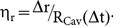
(2)


Here, the spatial overlap parameter represents the following scenarios: (i) η_r_<1: The subsequent laser pulse impinges the existing cavitation bubble inside the sample medium and (ii) η_r_>1: The second laser pulse is focused inside the sample medium next to the existing cavity.

These two overlap parameters were used as retrospective parameters for an optimum mutual comparison of the following results. They maintained the description of the basic interaction scenarios in water because using them in a surgical laser control would require free choice of repetition rate and pulse energy as well as the knowledge of exact LIOB threshold energy and rheology of the tissue.

## Results

### Determination of the LIOB threshold in water

Because different target materials were used the LIOB threshold was determined for water and each material. For this experimental setup (see Section 2.1) the measured single pulse breakdown threshold energy was E_th_ = 151±10 nJ (precision of determination <5%); this value refers to the laser focal spot within the glass cuvette and considers all the energy losses due to optical elements within the laser path. Thus, for given experimental parameters and under the assumption of a diffraction-limited spot diameter the dedicated fluence at the laser focus was about 5.02±0.33 J/cm^2^.

Experimentally, no significant difference could be found for the breakdown threshold in media with different rheological properties, e.g. gelatin solutions of various concentrations. For this reason, the pulse energies applied in the experiments presented here were related to the breakdown pulse energy within distilled water. All energy values in the following are given as multiples of this threshold value.

### Observable interaction mechanisms and its characteristic effects in water

**Figure 3:**
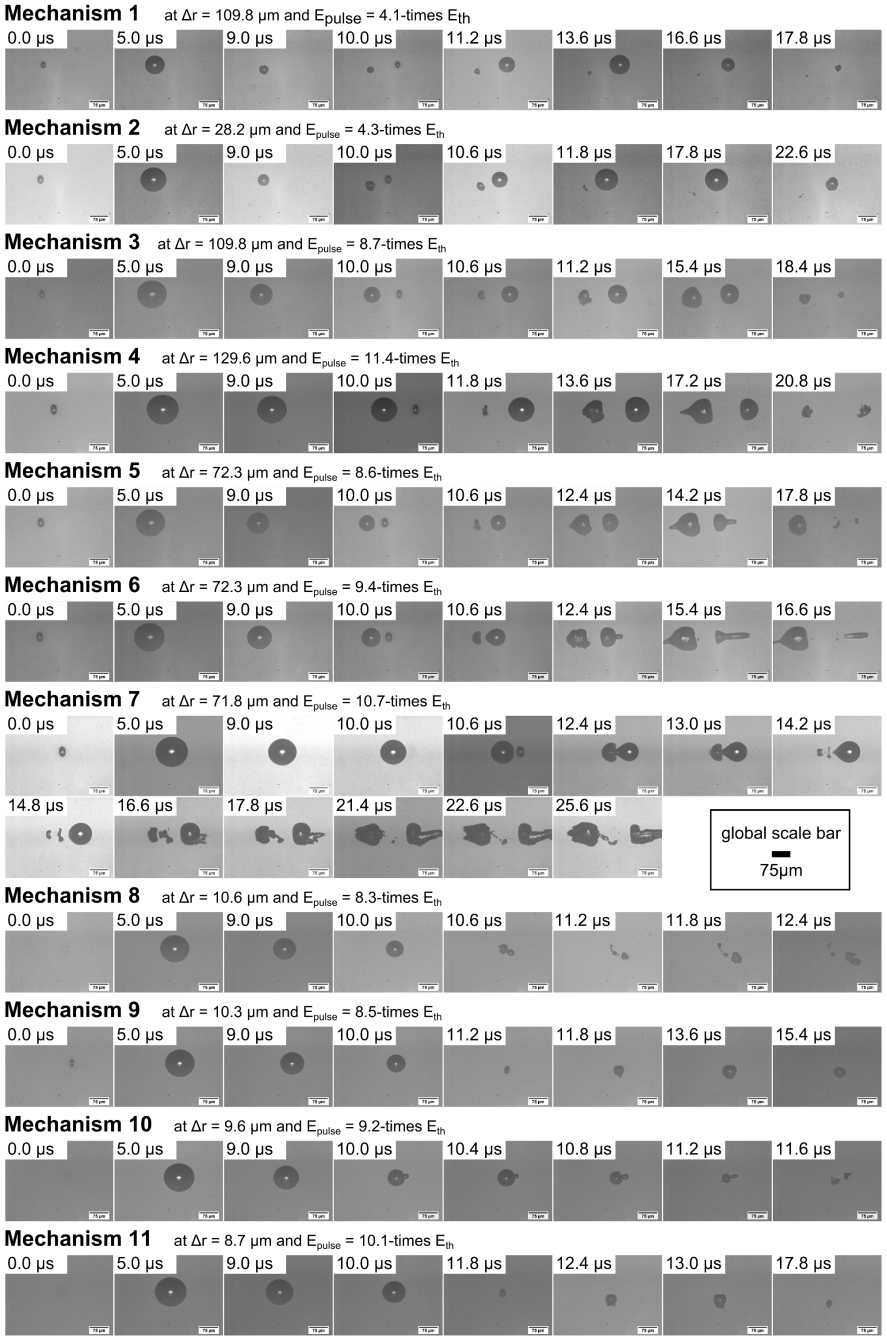
Cavitation bubble dynamics of different observable interaction mechanisms. The first cavitation bubble occurs at about 0.0 µs for every image series. Its single bubble dynamics is shown in two more frames at 5.0 µs and 9.0 µs. The second cavity with defined temporal and spatial separation appears at 10.0 µs next to the first one. Afterwards the dynamics of the cavitation bubble interaction is shown at selected points in time. A more detailed depiction with equidistant time steps can be seen in S2 Figure. Especially, the jet formation of interaction mechanism 7 is shown in Fig. 4 for the whole duration of oscillation and for the total jet length.

**Figure 4:**
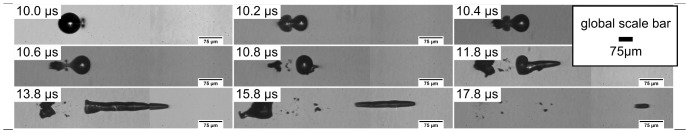
Detailed bubble dynamics of two cavities in the observable interaction mechanism 7. The parameters to observe mechanism 7 were here a focus separation of Δr = 71.8 µm and a laser pulse energy of E_pulse_ = 10.7-times E_th_. The image series begins with the occurrence of the second cavity at 10 µs. Afterwards, the dominating jet formation in laser scanning direction is shown with the overall jet length by composing two images covering different imaging regions within the cuvette at the same time delay. A more detailed time evolution of the effects and the whole dynamics in equidistant time steps is shown in the S3 Figure).

**Figure 5:**
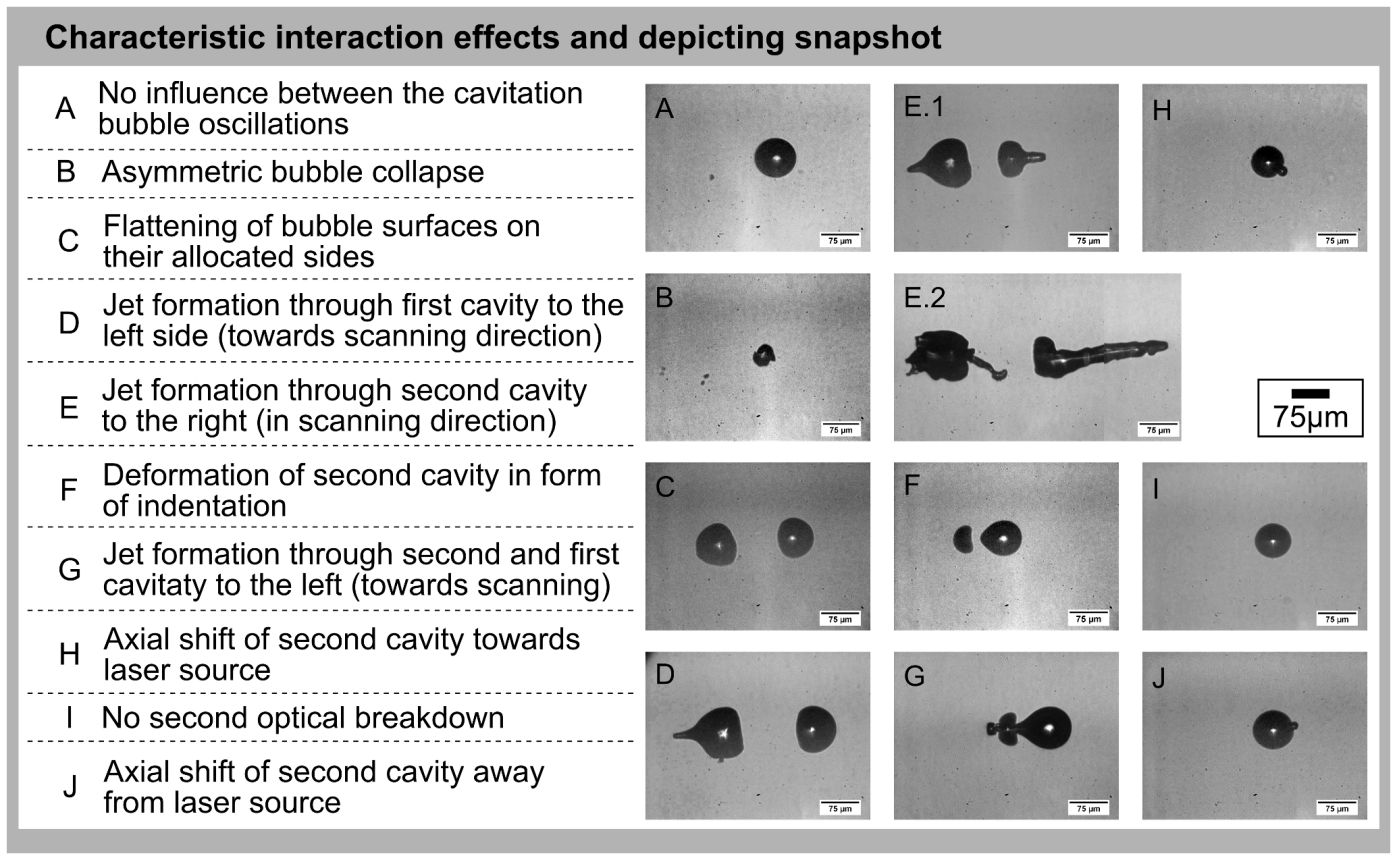
Characteristic interaction effects of two spatially and temporally separated laser pulses and their cavitation bubbles. The depicted interaction effects of two spatially as well as temporally separated laser pulses and their cavitation bubbles are clearly represented in this depiction. The characteristic effect is specified on the left side and shown as an example snapshot on the right. Effect E is shown in form of two different snapshots to reveal the diversity of jet characteristics and strength, respectively.

Inside water as sample medium, two temporally as well as spatially separated laser pulses showed a very complex interaction and hence different resulting bubble dynamics compared to the single bubble dynamics. For that reason the two-dimensional parameter space was analyzed regarding the various observable effects and mechanisms first. The different cavitation bubble dynamics are shown in Fig. 3 as series of 8 or more single pictures and, additionally, for mechanism 7 in Fig. 4. Here, starting at a time of 10.0 µs the depicted images are composed of two pictures taken at different imaging regions to cover the whole jet length. (A more detailed time evolution of the effects and the whole dynamics in equidistant time steps is shown in the S2 and S3 Figures). Depending on the scanning velocity, which defined the spatial focus separation Δr, and on the pulse energy comparatively to the breakdown threshold (encoding the temporal overlap) a number of characteristic interaction effects was detected (confer to dynamics in Fig. 3). In Fig. 5 a depiction is shown listing the ten different, overall observable interaction effects. The snapshots of characteristic interaction effects shown in Fig. 5 were selected from picture series of the whole oscillation dynamics.

**Figure 6:**
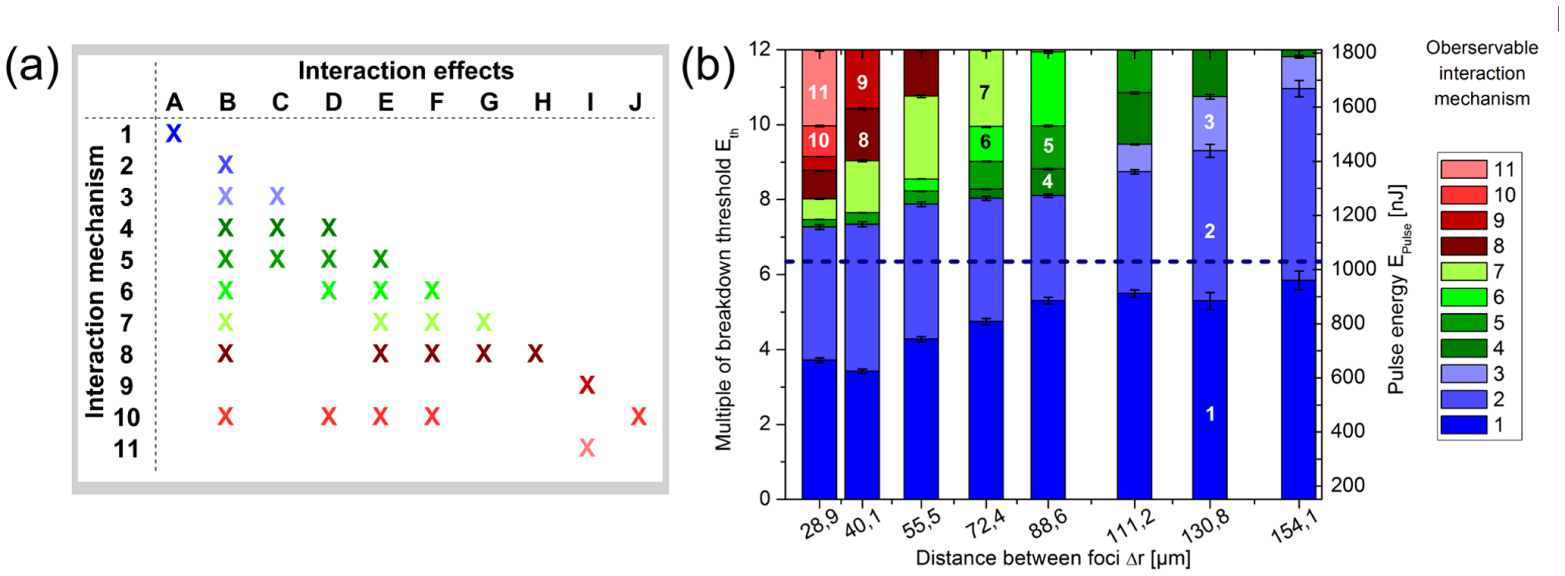
Observable interaction mechanisms of two spatially and temporally separated laser pulses and their cavitation bubbles. (**a**) Overview of occurring interaction effects A to J as part of the observable mechanisms 1 to 11. The depiction shows, which effects are combined as a superposition within each of the experimentally observable interaction mechanisms. (**b**) Bar diagram of observable interaction mechanism depending on the stepwise adjusted distance between the two foci Δr and the continuously varied applied pulse energy. Vertical lines mark the measured limit between two significantly different mechanisms. The error bars show the standard deviation resulting from the experimental measured pulse energy over 50 pulses as well as energy losses due to a vignetting of the scanning setup. The selected colors differentiate between weak interaction mechanisms (blue), strong interaction mechanisms within the scanning plane (green) and suppressed or axially medium-affecting interaction mechanisms (red). The dashed horizontal line denotes the applied pulse energy, above which the resulting cavitation bubble had a lifetime>10 µs.

Applying two laser pulses with a set of parameters for pulse energy and spot separation complex interaction mechanisms could be observed. In most of the cases, more than one of the interaction effects appeared as part of a mechanism (see Figs. 3, 5 and 6a); the concerned effects occurred one after another in the course of time. For example, if interaction mechanism 5 proceeded the effects B (asymmetric bubble collaps), C (flattening of bubble surfaces on allocated sides), D (jet formation through first cavity towards scanning direction), and E (jet formation through second cavity in scanning direction) could be observed (confer to dynamics in Fig. 3). Here, an assumed interaction mechanism corresponds to a significant change in appearing combination of interaction effects. The effects themselves showed a sufficient reproducibility regarding their manifestation for constant laser parameters (2 to 3 times per individual time point). The dependency between the resulting interaction mechanisms and the experimental parameters is shown in Fig. 6b for the 8 different analyzed distances between the two foci. Here, at the border of two mechanisms the standard deviation of pulse energy over 50 pulses as well as energy losses due to a vignetting of the scanning setup were indicated as error bars. As an example, for constant spot separation of 28.9 µm up to 3.7-times the breakdown threshold interaction mechanism 1 was observable. By further increasing the applied pulse energy up to 7.3-times threshold the effects of mechanism 5 appeared. Mechanism 6 occurred until a next significant change of effect combination at 7.5-times E_th_. Afterwards, mechanism 7 was observable at the focal volume up to 8.0-times breakdown threshold. A further increase in pulse energy led to mechanism 8 until 8.8-times and mechanism 9 up to 9.2-times threshold, respectively. Following this, mechanism 10 was observable up to 10.0-times E_th_. Afterwards, mechanism 11 appeared up to the maximum applied pulse energy of 12.0-times breakdown threshold. In turn, the occurring interaction mechanisms become weaker for larger focal distances as for a constant spot separation of 130.8 µm for example: Here, mechanism 1 was observable up to 5.3-times breakdown threshold. An increase in applied pulse energy led to mechanism 2 until 9.3-times E_th_. Afterwards, mechanism 3 appeared until 10.8-times breakdown threshold, while mechanism 4 was observable up to the maximum applied energy of 12.0-times threshold.

Overall, a very high temporal and spatial distance of subsequent pulses as for mechanism 1 led to a laser-material interaction based on single bubble disruption. Here, there was no impact of two succeeding pulses' bubbles on each other's dynamics. A high spatial overlap, which means the other extreme, and hence a pulse focusing into an existing cavity led to a decrease in laser energy absorption [27] and, partially, to an axial focus shift (see mechanisms 8 to 11). The mechanisms 4 to 7 in between showed a combination of highly complex interaction effects. For this high temporal overlap and a moderate spot separation of the second pulse with the first pulse's cavitation bubble liquid jets along the scanning axis were observable. The dashed horizontal line denotes the applied pulse energy, above which the resulting cavitation bubble had a lifetime>10 µs; that means for each parameter pair beyond this line the subsequent laser pulse was focused into the sample medium during the first oscillation cycle of the existing one.

**Figure 7:**
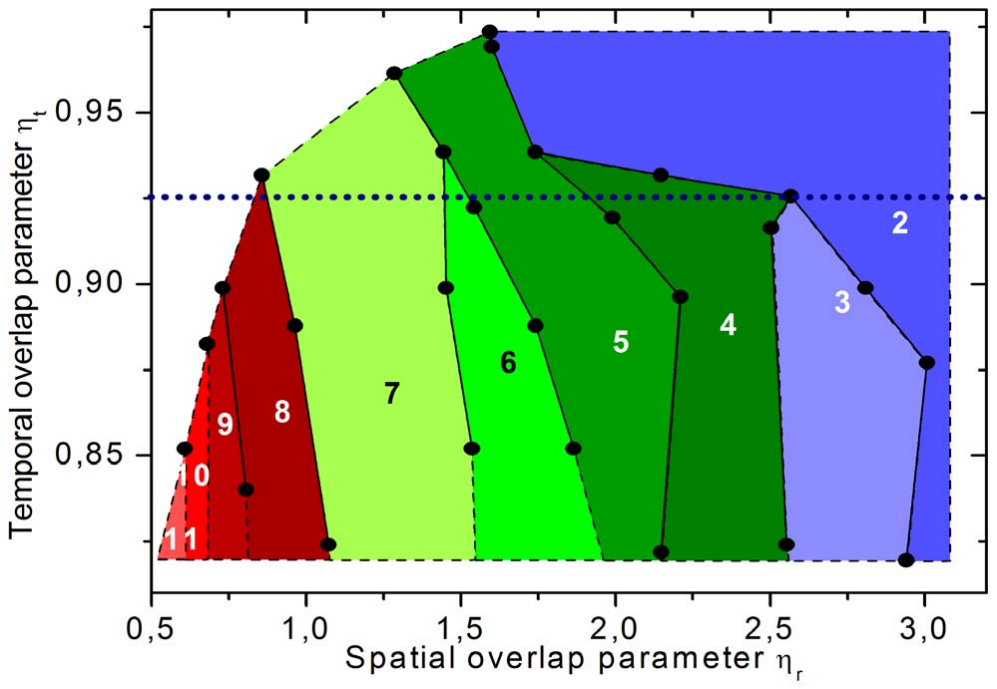
Color-coded map of the different interaction mechanisms in the effective parameter space of pulse overlap. The map includes the parameter space of spatial overlap parameter η_r_ and temporal overlap parameter η_t_. The dots mark the experimentally measured limit of different interaction mechanisms. For visual assistance they were connected by lines while the space in between was filled with the color belonging to the prevalent mechanism. The mechanisms are described in Fig. 4 regarding their combination of characteristic interaction effects.

Additionally, the temporal as well as spatial overlap parameters associated with each series of measurement were calculated. The representation of interaction mechanisms within this effectively analyzed parameter space is shown in Fig. 7. It can be seen that for a temporal overlap η_t_<0.925 (see dotted horizontal line in Fig. 7) there was a dependency of the interaction mechanism only on the spatial overlap parameter η_r_. This means that only the distance between the focal spot of the subsequent laser pulse and the first bubble's center of mass was essential for the resulting interaction mechanism: For a large overlap (η_t_<1, η_r_<1) and impinging an existing cavitation bubble with the subsequent laser pulse a further LIOB was suppressed on the one hand. On the other hand, an axial bubble shift with decreasing energy conversion efficiency appeared. The bubble collapse was asymmetric and there were liquid jets directed in surrounding medium which had an axial amount of mechanical impact. In contrast, for a temporal overlap (η_t_<1) combined with an increasing spatial separation between existing cavity and subsequent laser pulse (1<η_r_<2.5) effects with strong mechanical impact perpendicular to the optical axis of the laser could be observed. For this reason, the properties of this jet and their dependency on the pulse separation were studied in detail with water as sample medium; the results are shown in the following section.

### Analysis of the characteristics of the jets generated in scanning direction

**Figure 8:**
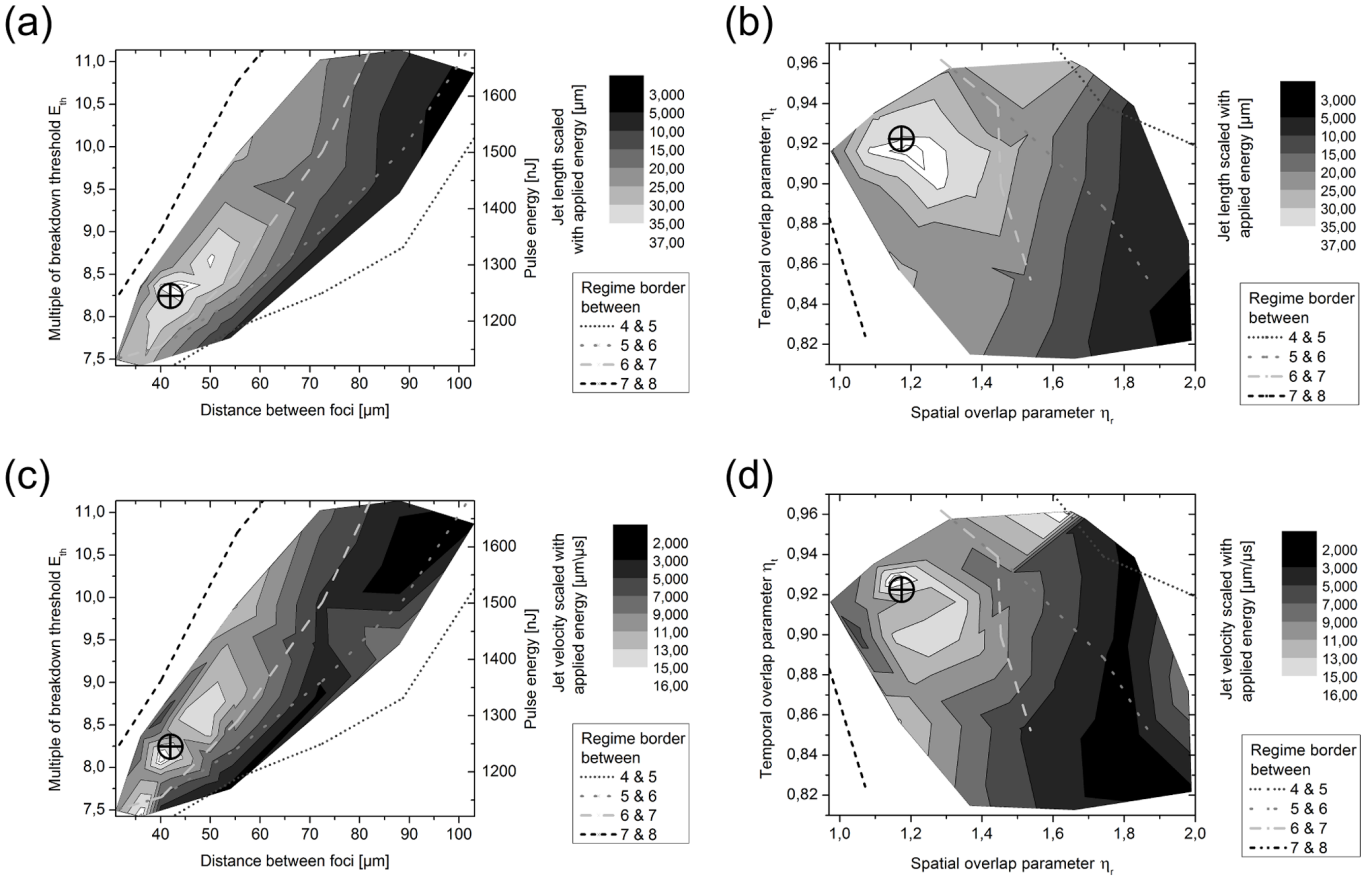
Contour depiction of the jet characteristics scaled with the applied laser pulse energy. Jet length within the parameter space of (**a**) focus separation and pulse energy (scaling the temporal overlap) as well as (**b**) spatial overlap parameter η_r_ and temporal overlap parameter η_t_, and jet velocity as a function of (**c**) focus separation and pulse energy as well as (**d**) the overlap parameters η_r_ and η_t_. The cross signs the maximum impact on the untreated medium (here water) at a maximum value for jet length and velocity at the same time. The dashed and dotted lines show supposed borders between the previously introduced interaction scenarios for visual assistance.

The strong water jet along the trajectory of laser scanning was observable within the interaction mechanisms 5 to 7 (see Figs. 3–4 and 6). The jet's properties which were affecting its influence on the untreated medium are the maximum jet length, and hence the range of impact, as well as its maximum velocity (regarding its operating momentum). These characteristic values were analyzed regarding their magnitude within the parameter space of pulse energy (coding the temporal overlap) and focus separation. The results of this examination can be seen as a contour plot in Fig. 8a and Fig. 8c. Furthermore, the Figs. 8b and 8d show an analogue depiction as a function of the overlap parameters. The grey-scale value maps the maximal length (Figs. 8a and 8b) and velocity, respectively (Figs. 8c and 8d). The four dashed and dotted lines depict assumed borders between the interaction mechanisms defined in Section 3.2. For a specification of the effective jet impact the dimensions were scaled with the applied laser pulse energy.

Additionally, in each graph of Fig. 8 a cross signs one value as an optimum parameter set which is located within mechanism 7. Here, the jet length as well as the jet velocity were as large as possible at the same time. For neglecting the scaling with applied pulse energy, the maximum jet velocity increased up to 135 m/s while the maximum jet length amounted to 309.5 µm. Here, the applied parameters corresponded to a focal separation of about 42 µm and a pulse energy of 8.3-times the breakdown threshold; consequently, the optimum overlap parameters were η_t_ = 0.92 and η_r_ = 1.17. Overall, the maximum jet length is equivalent to 5.3-times the maximum bubble radius R_max_ = 58 µm at the same pulse energy. Hence, assuming two pulses, which are applied with the optimum overlap parameters, the overall dissection length along the laser scanning axis is about 360 µm; this is 55% more than due to the single pulse cutting with η_t_>1.0 and η_r_ = 2.0 (for pulse energies far above the breakdown threshold as applied here).

### Interaction of two laser pulses in porcine gelatin and vitreous body as sample media

The dependency of the effects on the existing pulse-to-bubble overlap could be approved in porcine gelatin as well as vitreous body of enucleated porcine eyes. Here, the behavior of the bubble-to-bubble interaction was analyzed in another four sample media besides de-ionized water: porcine vitreous body as well as porcine gelatin in water solution of the concentrations 1%, 2%, and 5%.

**Figure 9:**
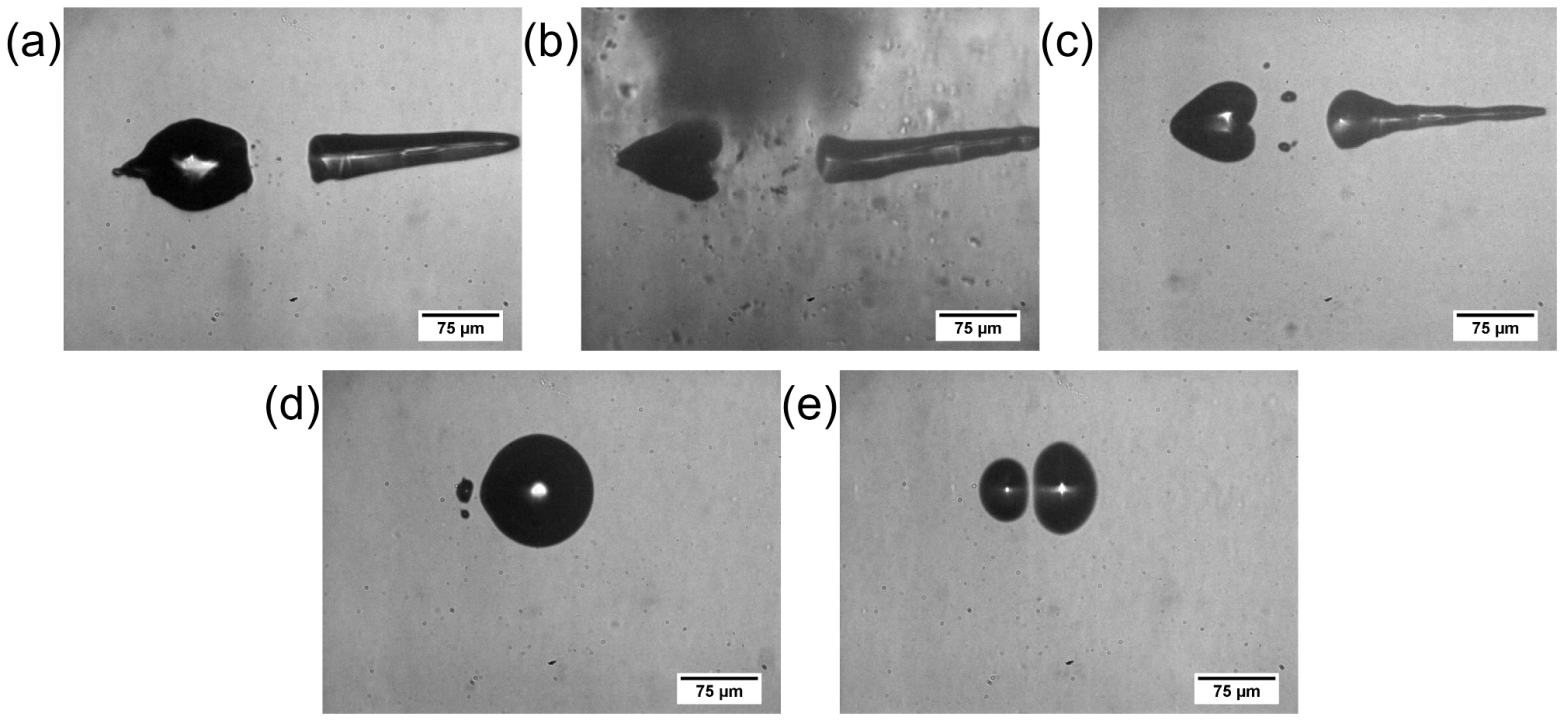
Snapshots of the bubble-to-bubble interaction within different sample media. Images show representative interactions after application of constant laser parameters in: (**a**) De-ionized water, (**b**) porcine vitreous body (increased artefacts due to inhomogeneities inside the biological tissue), (**c**) 1% gelatin solution, (**d**) 2% gelatin solution, and (**e**) 5% gelatin solution. The pulse energy E_pulse_ corresponds to 8.5-times breakdown threshold E_th_ in water and the focus separation confirms to Δx = 46.1 µm. All pictures are taken at Δt = 13.6 µs. While (a), (b), and (c) show the typical interaction effects of mechanism 7, in (c) and (d) mechanism 2 conveys.

To begin with, for focusing two subsequent fs-laser pulses with constant pulse energy E_pulse_ as well as spot separation Δr inside the different sample media, a change of occurring interaction mechanisms could be observed. For a pulse energy of 8.5-times the breakdown threshold E_th_ in water and a distance between the foci Δr = 38 µm a similar bubble oscillation appeared in water, vitreous body and the 1% gelatin solution (see Table 1). All interaction effects within these media belonged to mechanism 7: There was the jet through the first cavitation bubble to the left (towards laser scanning, characteristic effect D). Afterwards, the second jet formed in scanning direction (characteristic effect E) which is shown in Fig. 9 for all media at related parameters of E_pulse_ = 8.5-times E_th_ and Δr = 46.1 µm. While the jet properties in general were very similar for porcine vitreous body compared to de-ionized water, their reproducibility falled slightly due to the inhomogeneities within the biological tissue. This effect was clearly recognizable due to a comparison of the three images at each point of time during the whole bubble dynamics. As can be seen in Fig. 9b there are comparably large tissue structures within the vitreous body. These led to an increased variation of jet length as well as changes of the exact jet direction. In detail, in some single image there could be observed no jet at all. However, for 1% gelatin the maximum bubble radius R_max_ of the first cavity as well as the jet length decreased.

**Table 1:** Overview of the analyzed sample media with constant spot separation.

Sample medium	Multiple of E_th_	E_pulse_[nJ]	Δr[µm]	Spatial overlapparameter η_r_	Observablemechanism
De-ionized water	8.46	1221	38	./.	7
Vitreous body	8.46	1221	38	1.15	7
Gelatin 1%	8.46	1221	38	1.33	7
Gelatin 2%	8.46	1221	38	7.7	2
Gelatin 5%	8.46	1221	38	5.62	2

Overview of the analyzed sample media and the applied laser parameters pulse energy E_pulse_ (also in multiple of the threshold E_th_ for de-ionized water, the given values lie within the precision of the threshold determination) and spot separation Δr, which are constant here for all media. However, the resulting spatial overlap parameter η_r_ as well as the observable interaction mechanism vary for different mechanical properties.

In contrast, for the 2% and 5% gelatin the interaction mechanisms obviously changed: Instead of jet formation, which belongs to mechanism 7, only an asymmetric bubble collapse (characteristic effect B) was observable during the interaction oscillation (mechanisms 2). An overview of the parameters and the resulting mechanisms is shown in Table 1.

A possible reason for this behavior was a shift of the effective spatial overlap, which is described by the parameter η_r_ and can be found in Table 1 as well. Due to the decrease in maximum bubble radius R_max_ and lifetime T_c_ with increasing gelatin concentration the applied laser pulse energy did not suffice for achieving the same spatial overlap. Here, the temporal overlap parameter η_t_ was neglected for there was no influence on the interaction effects for η_t_<0.925 (see Section 3.2 and Fig. 7). While the spatial overlap parameters for vitreous body (η_r_ = 1.15) and 1% gelatin (η_r_ = 1.33) lied within the range of mechanism 7 for water (1.05<η_r_<1.5; see Fig. 7), the parameter was significantly increased for 2% gelatin (η_r_ = 7.7) and 5% gelatin (η_r_ = 5.62).

**Figure 10:**
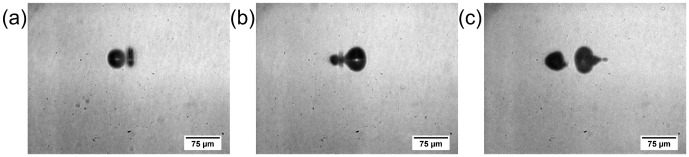
Single snapshots of the cavitation bubble interaction dynamics in a 5% porcine gelatin solution. The pulse energy is 10.5-times breakdown threshold in water and the focus separation confirms to 30.4 µm; the spatial overlap parameters is η_r_ = 1.41: (**a**) Formation of second cavity close to first one, (**b**) jet formation through first bubble, and (**c**) jet through right cavitation bubble along the direction of laser scanning.

Further confirmation was given by the experimental results presented in Table 2. Here, the range of the spatial overlap parameter η_r_ of water was specified for the interaction mechanisms 2 (η_r_>2.85), 7 (1.05<η_r_<1.5) and 8 (0.77<η_r_<1.05). For the other four analyzed media the experimentally applied laser parameters pulse energy E_pulse_ and spot separation Δr were adapted relatively to those applied to the sample medium water regarding the spatial overlap (η_t_<0.925 in all cases). This led to a compliance in the spatial overlap parameter η_r_, and furthermore, in the resulting interaction effects and mechanisms, respectively. For example, as for interaction mechanism 7 in water even in 5% gelatin solution the following effects were observable (e.g. Fig. 10): The generation of a further cavitation bubble close to the existing one (see Fig. 10a) led to jet formation through the latter one towards the laser scanning direction (see Fig. 10b, characteristic effect D). The following jet in scanning direction was clearly visible as well (see Fig. 10c, characteristic interaction effect E). It has to be noticed that the jet length again decreased with increasing gelatin concentration and hence scaled with rheological properties of the sample medium.

**Table 2:** Overview of the analyzed sample media with adapted spot separation.

Sample medium	Multipleof E_th_	E_pulse_[nJ]	Δr[µm]	Spatial overlapparameter η_r_	Observablemechanism
*De-ionized water*				*>2.85*	*2*
De-ionized water	3.71	521.3	29	5.22	2
Gelatin 2%	8.46	1221	38	7.7	2
Gelatin 5%	8.46	1221	38	5.62	2
*De-ionized water*				*1.05*–*1.5*	*7*
De-ionized water	7.46	1126	29	1.28	7
Vitreous body	8.46	1221	38	1.15	7
Gelatin 1%	8.46	1221	38	1.33	7
Gelatin 5%	10.5	1576	30.4	1.41	7
*De-ionized water*				*0.77*–*1.05*	*8*
De-ionized water	8.02	1231	29	0.856	8
Gelatin 2%	10.7	1602	30.4	0.825	8

Overview of the analyzed sample media and the applied laser parameters pulse energy E_pulse_ (also in multiple of the threshold E_th_ for de-ionized water, the given values lie within the precision of the threshold determination) and spot separation Δr. By adapting these for the different media spatial overlap parameters η_r_ within the same range as for water are achieved. In this case, the observable interaction mechanisms correspond.

In conclusion, only the spatial overlap of the subsequently focused laser pulse to the existing cavitation bubble seems to be responsible for the resulting effects of the bubble-to-bubble interaction. Overall, the overlap parameters are transferable to the other sample media, while the jet characteristics are modified by the mechanical properties.

### Interaction of a series of subsequent laser pulses in water

**Figure 11:**
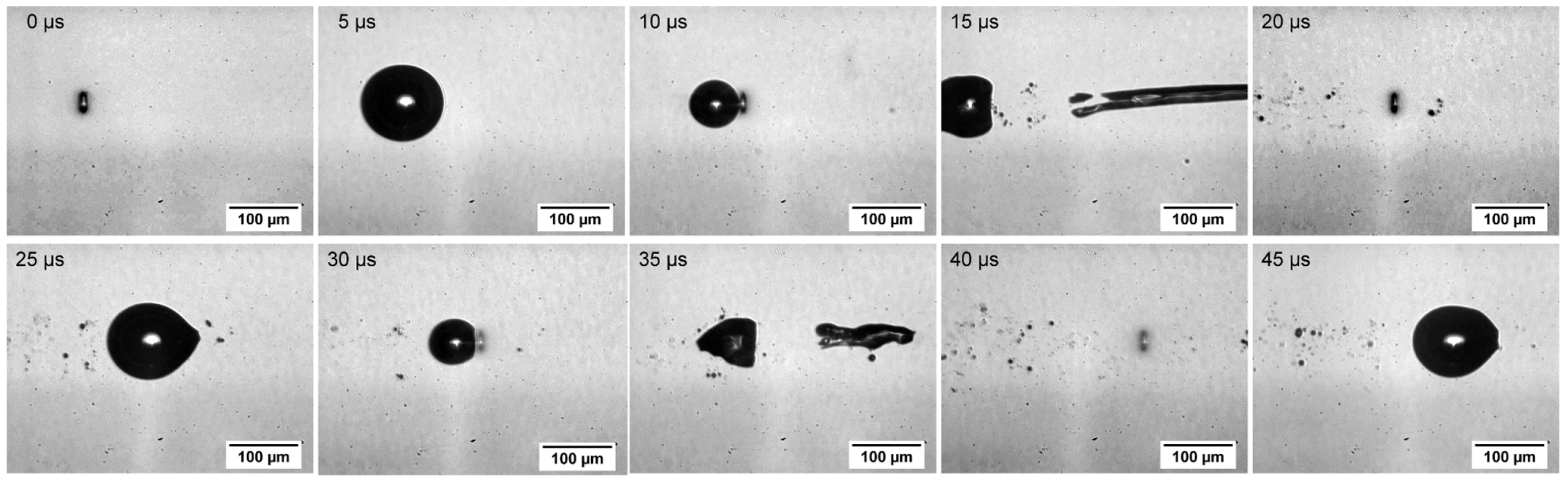
Cavitation bubble dynamics after five subsequent laser pulses in water. Single pictures of the cavitation bubble dynamics due to the application of five subsequent laser pulses using the optimum parameters for water (see Section 3.3). The second and fourth cavities lead to jet formation (see pictures at 15 and 35 µs). Due to the modified overlap by the previous interaction mechanisms the second jet is weaker.

In Fig. 11, extracted pictures of the time-resolved analysis of the interaction of five laser pulses in water as sample medium are shown. While the progress shows the former interaction of two laser pulses at the beginning (bubble dynamics shown up to 15 µs), at a time of 20 µs, 30 µs, and 40 µs the third till fifth pulse impinged the medium. Due to the interaction of the first two cavities (applied overlap correlates with the optimum overlap parameters in water, see Section 3.3) there was the strong jet formation to the right (see characteristic effect E). The jet led to a premature cavitation bubble collapse so that the third pulse (after 20 µs) impinged only persistent gas bubbles at the focal volume. This means a first modification of the resulting overlap between a cavitation bubble and the subsequent laser pulse; the temporal separation was constant. In consequence of the persistent liquid flow the third cavitation bubble showed a deformation to the right side but no jet (see picture at 25 µs). The fourth LIOB occurred at about 30 µs. Here, due to the deformation of the previous bubble there was another, but less strong modification in spatial overlap. Hence, another jet formed which was decreased in its propagation length with increased statistical variation. The last and fifth pulse hit again persistent gas bubbles and results in a cavity with the same deformation as the third one.

Overall, the application of a pulse train was associated with a modification of the occurring effects after the subsequent laser pulses. The reason is the clearly visible change in the effectively resulting overlap due to premature bubble collapse after jet formation and the persistent water streaming of jets which affects the subsequent pulses.

## Discussion

Here, we presented an analysis of the cavitation bubble dynamics of temporally and at the same time spatially separated fs-laser pulses using time-resolved photography. As a systematic investigation of behavior of bubble-to-pulse and bubble-to-bubble interaction due to an overlap the results are discussed regarding the dissection quality of future-generation high-repetition rate ophthalmic laser systems.

### Immersion-free fs-laser focusing unit for generating a LIOB

For the setup, which was used for the presented experiments, no special efforts were made to minimize the spherical aberrations; for example, working with a water-immersion objective would be beneficial in that case [3]. Additionally, the NA of the presented experiments is below the threshold of 0.9 for negligible nonlinear effects in beam propagation [3], [8], [14], [36]. Furthermore, the cavitation bubble diameter at the breakdown threshold is well below 1 µm as shown in [35]; hence it is beneath the system's optical resolution of the imaging setup. By performing a detection of the onset light, which is scattered by the cavity, instead of the bubble's shadow a more accurate determination of the LIOB threshold energy would be feasible [14], [35]. These are possible reasons why the corresponding fluence at the laser focus lies within the upper range of other findings in literature (see overview in [14] and [6], [9], [35]). However, for this precise determination of LIOB threshold was not the aim of the presented study the optically identified threshold energy is taken as an upper limit approximation of the exact value.

### Validity of the experimental results regarding the state of the art

In previous publications the influence of only temporally or only spatially, respectively, separated pulses and hence cavitation bubbles on the dissection efficiency and quality during laser surgery could be shown [15], [27]. Here, the spatial as well as temporal overlap of pulses and bubbles led to various interaction scenarios which formed a complex superposition of previously observed effects like for example jet formation. But principally, the former findings of only spatially [15], [18], [20]–[21], [24] or only temporally separated [26]–[27] pulses and cavitation bubbles, respectively, are in good agreement with the interaction effects presented here. After the systematic grouping of the effects within the parameter field of spatial and temporal overlap, which is shown in Section 3.2, these are discussed regarding their impact on the medical application: Mechanism 1 describes the well-known single pulse interaction with the sample medium (see Section 1). Therefore, it has been extensively studied experimentally as well as theoretically before [1], [3], [5]–[11].

### Predictive value of mechanisms with jet formation to ophthalmic fs-laser treatment

However, there is a close resemblance of the mechanisms 8 to 11 and the interaction of only temporal separated laser pulses [26]–[27]; the influence of the subsequent laser pulse is suppressed by the still existing cavitation bubble of the previous one. There is no or only a very low-efficient further optical breakdown which has no contribution to the aspired cutting process for the second laser pulse hits the oscillating cavity. As shown in [27] before, the laser energy transmission increases and though does the linear absorption of light at the retina. Hence, the laser energy will be partly or even completely lost for the cutting process itself. The effectiveness of the cutting process in terms of the used fluence as well as its precision is lowered and the influence of linear thermal effects at the retina grows. Additionally, an axial shift of the second cavitation bubble (see characteristic effects H and J) is accompanied by jet formation during the first cavity's collapse phase. In detail, these jets are directed along the bubble connection line and impinge the surrounding tissue. This means, they lead to an axial amount of mechanical impact which decreases the cutting precision and hence quality. Thus, for an optimization of precise tissue dissection with high-repetition rate fs-laser systems these effects should be necessarily avoided. Nevertheless, axial jetting could possibly imply an advantage for fs-laser cataract treatment, if there is any jet formation inside the hardening tissue of the crystalline lens [37].

From a medical point of view, possible positive impact could be ascribed to the mechanical effects in the direction of laser scanning; the two liquid jets as part of the mechanisms 4 to 7 for example might promote the cutting process. A jet propagating towards the laser scanning direction impinges medium which is already processed (see characteristic effects D and G). This effect could be used for cutting backward located tissue bridges. Certainly, if the jet is directed along the scanning axis (characteristic effect E) it hits and possibly dissects untreated tissue. And thus, less total energy could be applied. The exact amount of energy saving due to tissue cutting by jet formation has to be analyzed more detailed for pulse energies close to the breakdown threshold.

The effect of jet formation due to oscillating cavitation bubbles is well known for conditions like a border close to the bubble surface [38]–[43] or two only spatially separated cavitation bubbles [15], [18], [21], [24].

However, the NA of 0.65 used in this study lies within the typical order for corneal laser treatment. For applying the laser inside the crystalline lens or even at the anterior eye segment (e.g. inside the vitreous body) the focusing NA has to decrease significantly. In turn, this leads to an increased occurrence of nonlinear propagation effects as for example self-focusing and hence streak formation [44]. Additionally, the plasma volume would become deformed in a prolate way which then leads to non-spherical cavitation bubble dynamics. Hence, the interaction between oscillating cavities would become even more complex. Therefore, the results of the experiments presented here are restricted regarding their transferability to laser treatment of ophthalmic tissue beyond the cornea.

### Transferability of the results to more complex sample media and application of pulse trains

As in other publications it was observable that for increasing mechanical strength of the sample medium, as here due to higher gelatin concentration, the maximum bubble radius at the same applied laser pulse energy decreases [27], [31]–[34]. This is the reason, why the effective overlap of a subsequent laser pulse with the existing cavitation bubble decreased (increasing overlap parameter η_r_) at constant laser parameters (see Table 2). The effect could be compensated by increasing the pulse energy E_pulse_ or decreasing the spot separation Δr, respectively (see Section 3.4). In conclusion, these results show that the findings for water regarding the dependency on the spatial pulse overlap (see Section 3.2) are completely transferable to gelatin as well as porcine vitreous body as sample media: The dependency of the interaction mechanisms on the absolute magnitude of the overlap parameters persists. Nevertheless, transfer of the results to cornea or lens tissue is as mentioned before only partly possible. Besides the influence of numerical aperture, inhomogeneities within the biological tissue lead to a significantly reduced reproducibility of the cavitation bubble dynamics; time-resolved photography as detection method is no longer appropriate. Therefore, an analysis of single events inside these solid inhomogeneous media by a high-speed photographic approach is necessary.

Furthermore, the results of an applied pulse train (see Section 3.5) showed a good consistency with the interaction of two laser pulses. Even if there was an increase of the number of pulses and thus complexity, the series of measurements confirmed that only the temporal η_t_ and especially spatial overlap η_r_ (for η_t_<0.925) with the previous pulse rules the upcoming interaction mechanism. Hence, it could be shown with these experiments that the results of the simplified two-pulse interaction (see Section 3.2 and 3.3) are very beneficial for evaluating and optimizing the cutting effect of high-repetition rate fs-laser systems. Based on these results, further experiments are desperately needed leading to an increase in understanding of the transferability to other media; especially anisotropic biological tissue like the crystalline lens or cornea are of great interest regarding the final medical application. Furthermore, for medical applications of laser light inside the crystalline lens or even the vitreous body a variation of focusing NA during experiments has to be taken into account additionally [45].

### Outlook

The presented results show possible phenomena occurring during laser surgery due to spatially and temporally varying cavitation bubble-pulse overlap during laser scanning. However, dissection of tissue during minimally invasive ophthalmic laser surgery is ideally performed with pulse energies slightly above the breakdown threshold. In this case, a fs-laser induced cavitation bubble oscillates for some microseconds with a radius in the range of up to some micrometers. For the moment, the fundamental finding of transferability presented here allows for predicting the interaction behavior of subsequent laser pulses with existing cavitation bubbles at pulse energies close to the LIOB threshold. To confirm this assumption a thorough coverage of more realistic pulse energies combined with a high-repetition rate laser system should be the aim of further studies. Consequently, the dependency of the critical laser parameters on the repetition rate as well as on the scanning velocity will be achieved for optimizing the dissection quality of high-repetition rate fs-laser systems. Additionally, the transferability to cornea or crystalline lens should be investigated by high-speed photographic studies within these media and taking into account the decrease of NA during laser surgery inside the anterior part of the eye.

## Conclusions

Tissue cutting by scanning high-repetition rate fs-lasers might lead to a spatial and temporal overlap of cavitation bubble and focusing volume of subsequent laser pulses. This study shows that a high overlap has to be avoided in order to increase photodisruption efficiency or axial precision and minimize unwanted side effects. Thus, a minimally invasive procedure may be ensured.

Bubble-to-bubble interactions and, in particular, jets perpendicular to the axial laser direction as observed in this study are potentially useful to disrupt remaining tissue bridges on the backside of laser focus as well as cutting untreated medium in front of the actual laser position. Here, a balance between higher pulse energy necessary for strong jets and the general aim to reduce pulse energy has to be found. The ideal scanner-laser combination and synchronization has to be determined to really benefit from the high-repetition rate. This study identified the overlap parameters as crucial for an optimized jet forming in terms of its length as well as velocity. In any ophthalmic fs-laser surgery setup, these overlap parameters would have to be determined empirically starting with the individual LIOB energy threshold value. In general, more flexibility in temporal separation of the pulse (pulse on demand) could be advantageous on the side of the laser sources.

Furthermore, the findings for jets in water could be translated into the sample media porcine gelatin and porcine vitreous body. In corneal and lens tissue the rheology is more inhomogeneous and a robust utilization of jet forming for the cutting process might be challenging. For reaching a high precision a parameter choice inducing jets with axial components should be avoided. Future studies should concentrate on finding parameters which are robust against typical rheological and LIOB threshold variations as they occur in real tissue.

## Supporting Information

S1 Figure **Exemplary determination of jet velocity by analyzing the temporal development of jet length.** The depiction shows the temporal evolution of the jet length through the second cavity in laser scanning direction (characteristic effect E) for a spot separation of Δr  =  44 µm and three laser pulse energies between 8.3-times and 10.4-times breakdown threshold. The second cavity occurs at 10.0 µs inside water as sample medium. The error bars indicate the standard deviation over two replications per time step. Furthermore, the data was fitted by an asymptotical function. The jet velocity complies with the curve slope.
(TIF)

S2 Figure **Detailed cavitation bubble dynamics of different observable interaction mechanisms.** The first cavitation bubble occurs at about 0.0 µs for every image series. Its single bubble dynamics is shown in equidistant time steps of 1.0 µs until 10.0 µs and time steps of 0.6 µs afterwards.
(TIF)

S3 Figure **Bubble dynamics of two cavities in the observable interaction mechanism 7 in equidistant time steps.** The image series begins with the occurrence of the second cavity after a time delay of 10 µs. The parameters were a focus separation of Δr = 71.8 µm and a laser pulse energy of E_pulse_ = 10.7-times E_th_. The cavitation bubble interaction in form of jet formation in laser scanning direction is shown with the overall jet length by composing two images covering different imaging regions within the cuvette at the same time delay. Here, the time step between subsequent total images is 0.2 µs for the initial bubble interaction and 1.0 µs for the jet dynamics.
(TIF)
