# Correction: Interaction Mechanisms of Cavitation Bubbles Induced by Spatially and Temporally Separated fs-Laser Pulses

**DOI:** 10.1371/journal.pone.0117647

**Published:** 2015-02-11

**Authors:** The PLOS ONE Staff

Fig. 1 is an erroneous duplication of Fig. S1. The authors have provided a corrected version here.

**Figure 1:**
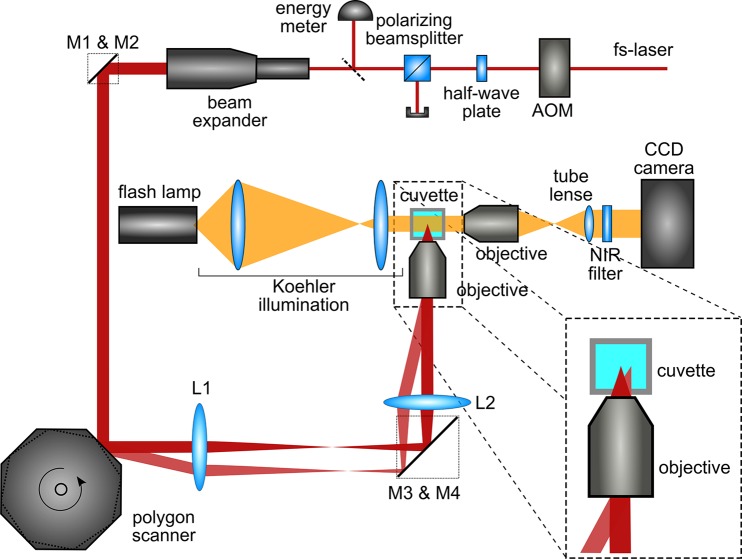
Schematic depiction of the experimental setup of the laser path (red) and the illumination path (orange). Single pulses of the fs-laser are selected by an acousto-optic modulator (AOM), half-wave plate and polarizing beam-splitter cube allow for laser power adjustment. Subsequent laser pulses are spatially separated via polygon scanner and a Keplerian telescope imaging (see also magnified image detail). The focal region inside the sample medium-filled cuvette is illuminated homogeneously by Koehler illumination and a magnified image of the cavitation bubble is reproduced on the chip of the CCD camera.
