# Supplementary figures and images for: Interaction Mechanisms of Cavitation Bubbles Induced by Spatially and Temporally Separated fs-Laser Pulses
Source: PLoS One. 2014 Dec 11;9(12):e114437. doi: 10.1371/journal.pone.0114437 (PMC4263672; doi:10.1371/journal.pone.0114437)

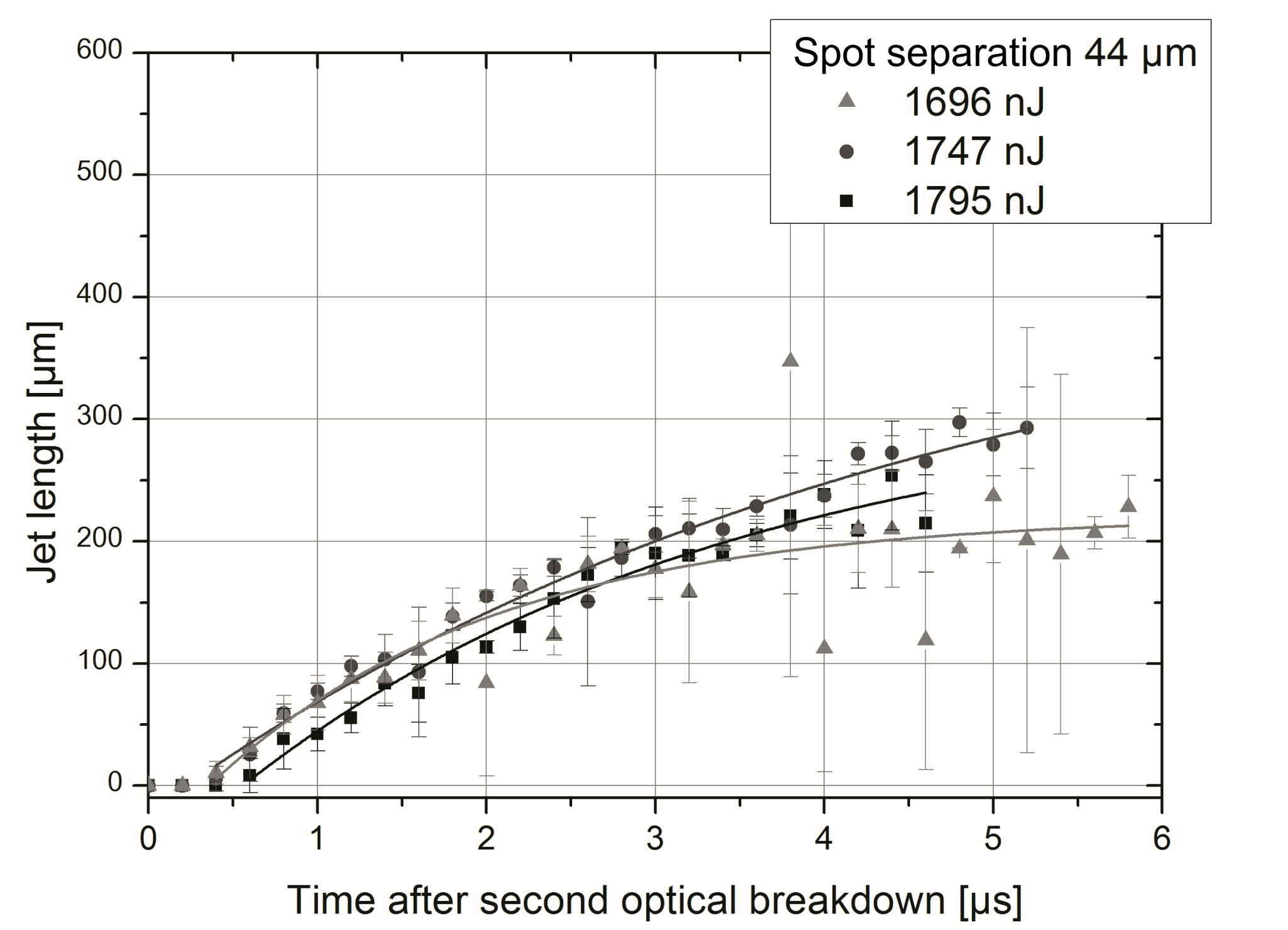

Supplement: S1 Figure — Exemplary determination of jet velocity by analyzing the temporal development of jet length. The depiction shows the temporal evolution of the jet length through the second cavity in laser scanning direction (characteristic effect E) for a spot separation of Δr = 44 µm and three laser pulse energies between 8.3-times and 10.4-times breakdown threshold. The second cavity occurs at 10.0 µs inside water as sample medium. The error bars indicate the standard deviation over two replications per time step. Furthermore, the data was fitted by an asymptotical function. The jet velocity complies with the curve slope. (TIF) [file pone.0114437.s001.tif]

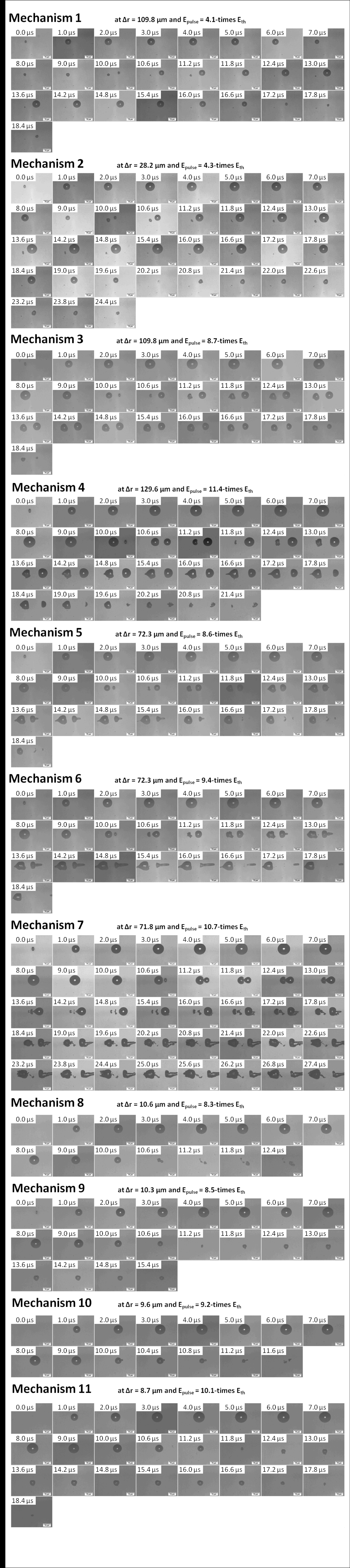

Supplement: S2 Figure — Detailed cavitation bubble dynamics of different observable interaction mechanisms. The first cavitation bubble occurs at about 0.0 µs for every image series. Its single bubble dynamics is shown in equidistant time steps of 1.0 µs until 10.0 µs and time steps of 0.6 µs afterwards. (TIF) [file pone.0114437.s002.tif]

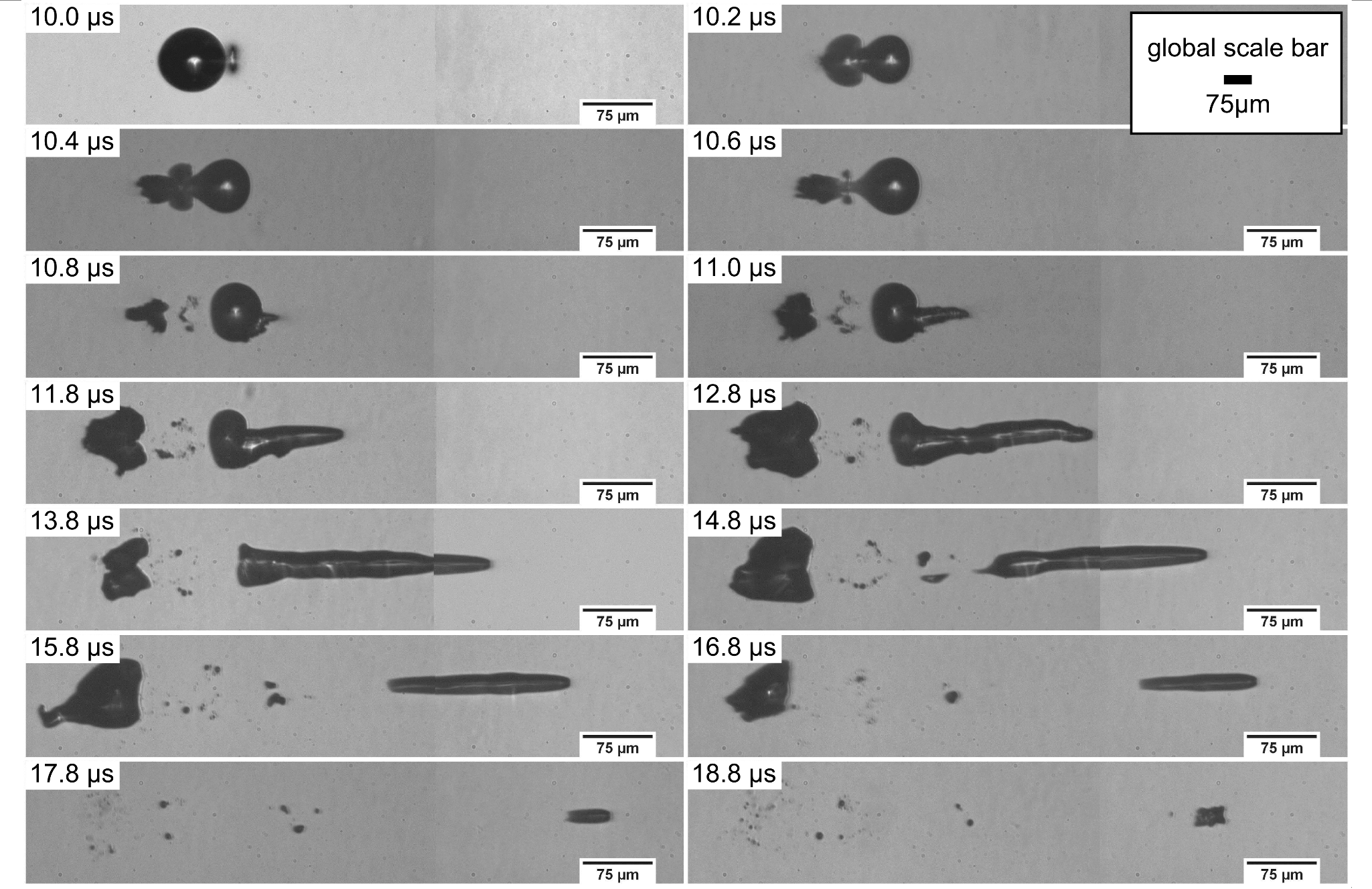

Supplement: S3 Figure — Bubble dynamics of two cavities in the observable interaction mechanism 7 in equidistant time steps. The image series begins with the occurrence of the second cavity after a time delay of 10 µs. The parameters were a focus separation of Δr = 71.8 µm and a laser pulse energy of Epulse = 10.7-times Eth. The cavitation bubble interaction in form of jet formation in laser scanning direction is shown with the overall jet length by composing two images covering different imaging regions within the cuvette at the same time delay. Here, the time step between subsequent total images is 0.2 µs for the initial bubble interaction and 1.0 µs for the jet dynamics. (TIF) [file pone.0114437.s003.tif]
